# Effect of children's shoes on gait: a systematic review and meta-analysis

**DOI:** 10.1186/1757-1146-4-3

**Published:** 2011-01-18

**Authors:** Caleb Wegener, Adrienne E Hunt, Benedicte Vanwanseele, Joshua Burns, Richard M Smith

**Affiliations:** 1 Discipline of Exercise and Sports Science, Faculty of Health Sciences, The University of Sydney, Cumberland Campus, PO Box 170, Lidcombe, 1825, NSW, Australia; 2 Faculty of Health Sciences, The University of Sydney/Institute for Neuroscience and Muscle Research, The Children's Hospital at Westmead, Locked Bag 4001 Westmead, NSW, 2145, Australia

## Abstract

**Background:**

The effect of footwear on the gait of children is poorly understood. This systematic review synthesises the evidence of the biomechanical effects of shoes on children during walking and running.

**Methods:**

Study inclusion criteria were: barefoot and shod conditions; healthy children aged ≤ 16 years; sample size of n > 1. Novelty footwear was excluded. Studies were located by online database-searching, hand-searching and contact with experts. Two authors selected studies and assessed study methodology using the Quality Index. Meta-analysis of continuous variables for homogeneous studies was undertaken using the inverse variance approach. Significance level was set at P < 0.05. Heterogeneity was measured by I^2^. Where I^2 ^> 25%, a random-effects model analysis was used and where I^2 ^< 25%, a fixed-effects model was used.

**Results:**

Eleven studies were included. Sample size ranged from 4-898. Median Quality Index was 20/32 (range 11-27). Five studies randomised shoe order, six studies standardised footwear. Shod walking increased: velocity, step length, step time, base of support, double-support time, stance time, time to toe-off, sagittal tibia-rearfoot range of motion (ROM), sagittal tibia-foot ROM, ankle max-plantarflexion, Ankle ROM, foot lift to max-plantarflexion, 'subtalar' rotation ROM, knee sagittal ROM and tibialis anterior activity. Shod walking decreased: cadence, single-support time, ankle max-dorsiflexion, ankle at foot-lift, hallux ROM, arch length change, foot torsion, forefoot supination, forefoot width and midfoot ROM in all planes. Shod running decreased: long axis maximum tibial-acceleration, shock-wave transmission as a ratio of maximum tibial-acceleration, ankle plantarflexion at foot strike, knee angular velocity and tibial swing velocity. No variables increased during shod running.

**Conclusions:**

Shoes affect the gait of children. With shoes, children walk faster by taking longer steps with greater ankle and knee motion and increased tibialis anterior activity. Shoes reduce foot motion and increase the support phases of the gait cycle. During running, shoes reduce swing phase leg speed, attenuate some shock and encourage a rearfoot strike pattern. The long-term effect of these changes on growth and development are currently unknown. The impact of footwear on gait should be considered when assessing the paediatric patient and evaluating the effect of shoe or in-shoe interventions.

## Background

Parents, health professionals and shoe manufacturers assume that children's shoes do not impede normal foot function or motor development. While it has long been thought that poorly designed and fitted shoes contribute to paediatric foot and toe deformity [1], empirical evidence of specific effects of shoes is equivocal. For example, cross-sectional studies suggest that children who usually wear shoes have a lower medial longitudinal arch than children who habitually go barefoot [2,3]. However, prospective studies have concluded that the medial longitudinal arch develops naturally and independently of footwear [4,5].

There is an existing body of literature on the biomechanical effects of shoes on the gait patterns of children. These effects are described according to the breadth of biomechanical variables including: spatio-temporal (relating to space and time); kinematics (relating to movement); kinetics (relating to external force and motion); electromyography (EMG) (muscle function) and plantar pressure [6]. While a number of studies have investigated specific variables within these categories [7-10], there is no recent cohesive review assimilating the known biomechanical effects of shoes on the gait of children. Of the two previously published reviews of the effects of children's shoes, one was published in 1991 [11] and the other focused only on children's sports shoes [12]. These reviews did not focus on the gait of children but rather on foot development, foot deformity, corrective shoes, foot anthropometry and the design requirements of shoes [11,12].

A systematic review updating the biomechanics literature would assist in identifying the effects of shoes on all aspects of children's gait. Such information will assist in the clinical assessment of paediatric shoe and in-shoe interventions, guide the development of children's shoes and assist in directing future research. The aim of this systematic review was to evaluate the evidence for biomechanical effects of shoes on walking and running gait, compared to barefoot in healthy children.

## Methods

### Inclusion and exclusion criteria

Inclusion and exclusion criteria for this study were determined *a priori*. Inclusion criteria were: children aged ≤ 16 years; barefoot and shod gait compared in a randomised or non-randomised order; healthy children described as developing normally and without pathology; a sample size of n > 1. Exclusion criteria were: novelty types of footwear such as roller skates or shoes with cleats; an evaluation of only foot orthoses, arch supports or innersoles.

### Search strategy

To identify relevant studies from online databases, the following search terms were truncated and adapted: shoe, footwear, shod, child, kid, p[a]ediatric, toddler, adolescent, infant, gait, walk, jog, run, ambula[te]tion. Database Medical Subject Headings (MeSH) terms were also used in seven of the nine databases (Medline, EMBASE, CINAHL, The Cochrane Library, AMED, EBM reviews, Sports Discus). Electronic databases searched were: MEDLINE (1950 to June 2010), EMBASE (1966 to June 2010), CINAHL (1967 to June 2010), The Cochrane Library (Second quarter 2010), Web of Science (1900 to June 2010), AMED (1985 to June 2010), EBM reviews (June 2010), SPORTDiscus (1790 to June 2010), Google Scholar (June 2010). Hand-searching was also undertaken of selected biomechanics journals, conference proceedings and reference lists of articles. To reduce publication bias, where studies with non significant findings are less likely to have been published [13], experts in the field were contacted to identify unpublished data. No restrictions were applied to year, language or publication type. One author undertook all searches in September 2009. Searches were updated in June 2010.

Two review authors determined independently from the title and abstract whether a study could be included. The full text was reviewed for clarification when required. Difference of opinion was resolved by discussion until consensus was achieved. Failing consensus, the opinion of a third author was sought.

### Quality assessment

The methodological quality of selected studies was assessed using the Quality Index [14]. The Quality Index is a validated and reliable checklist designed for the evaluation of randomised and non-randomised studies of health care interventions [14]. In the absence of a quality assessment tool designed for biomechanics studies, the Quality Index was considered appropriate in rigour with shoes treated as the 'health intervention'. A total score of 32 is possible across 27 items organised into 5 subscales: 10 items assessed study reporting (including reporting of study objectives, outcomes, participants characteristics, interventions, confounders, findings, adverse events and probability); 3 items assessed external validity (the ability to generalise the results); 7 items assessed internal validity selection bias (bias in the measurement of the intervention); 6 items assessed internal validity confounding (bias in the selection of study participants); 1 item assessed study power (to assesses if negative findings from a study could be due to chance).

Methodological quality of a study was assessed independently by two reviewers when published in English. The methodological quality of one study published in German [15] was assessed by a single author fluent in German. Rating for each item on the Quality Index was agreed by discussion.

### Data extraction

Data were extracted from studies written in English by one review author and from studies written in German by a second review author. Study authors were contacted for additional information, as required. Extracted data were checked by another review author. Shoe type was classified according to the Footwear Assessment Form [16]. If no information regarding the type of shoe investigated was attainable, the term 'unknown' was used.

### Statistical analysis

Meta-analysis was undertaken of homogenous studies where appropriate data were attainable. Mean differences, 95% confidence intervals and effect sizes were calculated. All analyses were undertaken in Review Manager 5.0 (The Cochrane Collaboration, Copenhagen, Denmark) using the inverse variance statistical method to calculate mean differences and 95% confidence intervals (CI) for continuous variables. This conservative technique assumes participant independence between the barefoot and shod groups, therefore increasing the confidence interval [13]. In biomechanical studies the standard practice has been to report the mean and standard deviation/error for the intervention and the control conditions, rather than reporting change scores between intervention and control conditions and change score standard deviation/error. This reporting practice prohibits the application of less conservative statistical techniques.

Statistical heterogeneity of included studies was assessed to determine if differences in results between studies included in the review were due to chance alone or study design. The quantity *I*^2 ^was utilised to assess statistical heterogeneity, where *I*^2 ^values of 25%, 50% and 75% represented low, moderate and high heterogeneity, respectively [17]. Where *I*^2 ^was greater than 25%, a random effects model analysis was used. Where *I*^2 ^was less than 25%, a fixed-effects model was used. When necessary, reported measures were converted to standard units, and standard errors were converted to standard deviations. Results were considered statistically significant if *P *< 0.05.

## Results

### Search results

**Figure 1:**
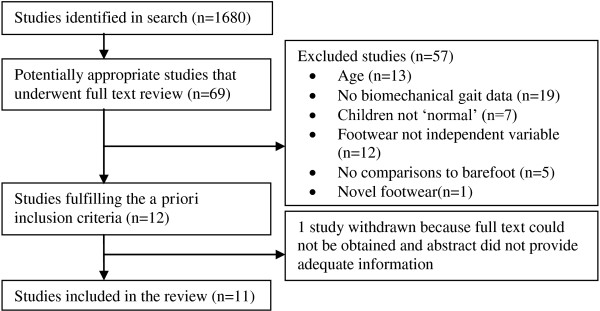
**Search and selection process for the review studies**.

Eleven studies met the inclusion criteria. The search and selection process is described in Figure 1. Nine papers were located through searching of online databases. Contact with known experts in the field located two additional unpublished research papers. An English translation of an abstract published in German indicated that the study met the criteria; however, the German text did not report a comparison between barefoot and shoes, making it ineligible for the review [18]. One unpublished thesis [19], was withdrawn from the review since the abstract provided insufficient data and the author was unable to be contacted for further data.

### Study quality

The median score for the Quality Index was 20 out of 32 (range 11-27 out of 32) (Table 1). In no study were participants blinded to the shoe interventions. In five studies the order of interventions was randomised [9,20-23].

**Table 1:** Methodological quality of the studies included in the review as assessed by the Quality Index

Author	Reporting (score/11)	External validity (score/3)	Bias (score/7)	Confounding (score/6)	Power (score/5)	Total (score/32)
Alcantara et al. [21]	7	1	4	1	4	17

Kristen et al. [15]	7	1	5	2	5	20

Lieberman et al. [25]	5	1	4	4	5	19

Lythgo et al. [7]	8	3	4	4	5	24

Moreno-Hernandez et al.[10]	7	1	4	3	5	20

Mueller et al. [22]	6	1	3	5	5	20

Oeffinger et al. [9]	6	1	5	1	5	18

Tazuke [26]	4	1	3	1	2	11

Wegener et al. [23]	8	1	5	4	5	23

Wilkinson et al. [20]	11	1	5	5	5	27

Wolf et al. [8]	8	1	5	2	5	21

### Participants

Data of children aged 1.6 to 15 years were evaluated from the included studies (Table 2). All but three studies in the review included children in middle childhood (ages 7 to 11 years) [15,20,24,25]. Boys accounted for 52% of participants.

**Table 2:** Description and methodological approach of studies included in the review

Author	Design	Sample size	Participants	Gait type	Shoe conditions	Outcome measure/s
Alcantara et al. [21]	Randomised repeated measures	8	4 girls and 4 boys, aged 7 to 14 years, mean age 10 years	run	barefoot/athletic/walking/walking	Kinetics

Kristen et al. [15]	Repeated measures	30	1.8-4.8 years	walk	barefoot/walking	Spatio-temporal, kinetics

Lieberman et al. [25]	Repeated measures	17	10 boys, 7 girls mean age 15 years	run	barefoot/unknown	Spatio- temporal kinematics,

Lythgo et al. [7]	Repeated measures	898	52% boys, aged 5-12 years	walk	barefoot/athletic	Spatio-temporal

Moreno-Hernandez et al.[10]	Repeated measures	61	31 girls, 30 boys, aged 10-13 years,	walk	barefoot/unknown	Spatio-temporal

Mueller et al. [22]	Randomised repeated measures	234	2-15 years, mean age 7.7 years	treadmill walk	barefoot/unknown	Electromyography

Oeffinger et al. [9]	Randomised repeated measures	14	8 females, 6 males aged 7-14 years	walk	barefoot/athletic	Spatio-temporal, kinematics

Tazuke [26]	Repeated measures	4	3 girls, 1 boy aged 8-13 years, mean age 10 years	run	barefoot/unknown	Spatio-temporal, kinematics

Wegener et al. [23]	Randomised repeated measures	20	8 girls, 12 boys aged 6-13 years, mean age 9 years	walk	barefoot/Oxford shoe	Spatio-temporal, kinematics

Wilkinson et al. [20]	Randomised repeated measures	31	17 girls, 14 boys, aged 1.1-2.7 years, mean age 1.6 years	walk	barefoot/athletic/Oxford shoe	Spatio-temporal, kinematics

Wolf et al. [8]	Repeated measures	18	8 girls, 10 boys aged 6-10 years, mean age 8 years	walk	barefoot/walking/flexible walking	Spatio-temporal, kinematics

### Shoe conditions

The shoe types that were commonly investigated were walking shoes (n = 5), athletic shoes (n = 4) and Oxford style footwear (n = 2) (Table 2). Four studies investigated multiple types of shoes [8,15,20,21]. Four studies did not describe the style of shoe investigated [10,22,25,26]. Five studies did not standardise the shoe worn [7,9,10,25,26].

### Description and methodological approach of included studies

The description and nature of the included studies are shown in Table 2. Nine studies investigated spatio-temporal variables, six studies investigated kinematic variables, two studies investigated kinetic variables and one study investigated EMG variables. Eight studies investigated variables in more than one type of biomechanical category. All but one study allowed participants to self-select gait velocity [22]. No studies reported monitoring gait velocity between conditions/trials. One study examined maximum sprinting velocity [26].

Wilkinson and colleagues [20] collected spatio-temporal variables from footprints of children walking barefoot and in two types of shoes. In order to reduce the variables examined, Wilkinson and co researchers [20] averaged all related measures to produce composite variables relating to time, angle of gait and stride/step length. The variable 'time' comprised the average of stride time, percent of time to foot lift, percent of time to maximum plantarflexion and the percent of time from foot lift to peak plantarflexion. The variable angle comprised the average of angle of gait relative to ipsilateral line of progression and angle of gait relative to the direction of gait. The variable length comprised the average of stride and step length. Wilkinson and co-investigators [20] also investigated the effect of footwear over time by reviewing children after a month of wearing randomly allocated athletic or Oxford style shoes. However, at the time of retesting analysis focused on comparison between shoes at the initial session and retest session and barefoot gait at the initial session and retest session. Therefore the retest data could not be included in this review.

Various methods were used across the six studies investigating kinematic variables [8,9,20,23,25,26]. Kinematics were investigated in three dimensions using multiple cameras in three studies [8,9,23] and in two dimensions using a single camera in three studies [20,25,26].

Biomechanical foot models also varied between studies. The foot was modelled as a single rigid body [9,20,25,26], and also as a multi-segmented structure [8,23]. Wegener and co-investigators [23] used a foot model of rearfoot (three calcaneal markers), forefoot (markers located at the navicular, 5^th ^metatarsal head and 1^st ^metatarsal head) and hallux segments (distal hallux marker). Motion was reported in three planes at the rearfoot complex and midfoot joints as flexion/extension, inversion/eversion and abduction/adduction in respect to the proximal segment, while resultant motion of the hallux was reported in two dimensions, primarily flexion/extension. Wolf and colleagues [8] used a modified Heidelberg foot model where the distance and rotations between the calcaneus and 1^st ^and 5^th ^metatarsal head markers were used to provide a measure of intrinsic foot function. The rotational angles within the foot were defined by the motion of 2D line-like segments around a perpendicular axis with respect to the proximal segment. This allowed for the examination of 10 variables to describe intrinsic foot function. Sagittal plane rearfoot motion was described by tibia-foot flexion, foot motion (rigid segment) relative to the tibia, and tibio-talar flexion, hindfoot motion relative to the tibia. Transverse plane foot motion was measured by foot rotation (complete foot motion relative to the tibia) and foot torsion (forefoot motion relative to the rearfoot). Frontal plane foot motion was described by 'subtalar' rotation (hindfoot motion relative to the tibia) and forefoot supination (forefoot motion relative to the ankle). Arch function was described by the change in distance between the medial calcaneal marker and 1^st ^metatarsal marker. Change in forefoot width was described by the distance between the 1^st ^and 5^th ^metatarsal markers. Foot progression angle was described by the orientation of the long foot axis relative to the direction of gait. Hallux sagittal plane motion (relative to the forefoot) was also described.

In addition to kinematics, information was obtained from kinetics and electromyography. Kinetics were investigated from force platform data in two studies [15,21] and from a tibial mounted accelerometer in one study [21]. EMG amplitude of the tibialis anterior, peroneus longus, and medial gastrocnemius during treadmill walking was investigated using surface electrodes [22].

### Spatio-temporal findings

The findings for mean difference, 95% CI, statistical significance, weighting and heterogeneity of walking spatio-temporal variables are presented in Table 3. Additional walking spatio-temporal details, including barefoot and shod values for each study, are reported in Additional File 1. Compared to barefoot walking, shod walking resulted in: increased walking velocity; longer stride length; longer step length; increased stride time; increased step time; decreased cadence; wider base of support; later toe-off time during the gait cycle; increased double support time; decreased single support; and longer stance time.

**Table 3:** Mean differences and statistical significance for spatio-temporal variables for shod and barefoot walking.

Variable	Shoe Condition	Authors	n	Weighting	Mean difference [95%CI]	Statistical significance: z Score (P)	**Heterogeneity: *I***^**2**^**%**
Velocity (m/s)	Athletic	Lythgo et al. [7]*	898	94.0%	0.07 [0.06, 0.09]	-	98%
	Unknown	Moreno-Hernandez et al.[10]	61	2.2%	0.05 [-0.01, 0.12]	-	-
	Athletic	Oeffinger et al. [9]	14	0.8%	0.04 [-0.08, 0.16]	-	-
	Oxford	Wegener et al.[23]	20	0.9%	0.03 [-0.08, 0.14]	-	-
	Walking	Wolf et al. [8]	18	1.4%	-0.01 [-0.10, 0.08]	-	-
	Combined	Pooled effect	1011	100.0%	0.07 [0.06, 0.08]	12.97 (P < 0.00001)	97%
	Walking (greater flexibility)	Wolf et al. [8]	18	100.0%	0.02 [-0.07, 0.11]	0.41 (P = 0.68)	N/A

Stride length (m)	Athletic	Lythgo et al. [7]*	781	97.60%	0.11 [0.11, 0.12]	-	97%
	Unknown	Moreno-Hernandez et al.[10]	61	1.10%	0.07 [0.02, 0.12]	-	-
	Athletic	Oeffinger et al. [9]	14	0.30%	0.12 [0.02, 0.21]	-	-
	Oxford	Wegener et al. [23]	20	0.20%	0.11 [0.00, 0.22]	-	-
	Walking	Wolf et al. [8]	18	0.70%	0.07 [0.01, 0.13]	-	-
	Combined	Pooled effect	894	100.0%	0.11 [0.10, 0.12]	40.49 (P < 0.00001)	93%
	Walking (greater flexibility)	Wolf et al. [8]	18	100.0%	0.06 [-0.01, 0.13]	1.71 (P = 0.09)	N/A

Step length (%)	Walking	Kristen et al. [15]	30	6.2%	0.20 [-2.26, 2.66]	-	-
	Athletic	Lythgo et al. [7]*	781	87.5%	9.69 [8.77, 10.61]	-	100%
	Unknown	Moreno-Hernandez et al.[10]	61	6.3%	6.57 [4.14, 8.99]	-	-
	Combined	Pooled effect	872	100.0%	8.90 [8.04, 9.77]	20.16 (P < 0.00001)	100%

Length (m)	Oxford	Wilkinson et al. [20]	31	100.0%	0.03 [-0.01, 0.07]	1.52 (P = 0.13)	N/A
	Athletic	Wilkinson et al. [20]	30	100.0%	0.04 [0.00, 0.07]	2.25 (P = 0.02)	N/A

Stride time (s)	Athletic	Lythgo et al. [7]*	790	94.0%	0.03 [0.02, 0.04]	-	99%
	Oxford	Wegener et al. [23]	20	2.6%	0.08 [0.03, 0.13]	-	-
	Walking	Wolf et al. [8]	18	3.4%	0.07 [0.03, 0.11]	-	-
	Combined	Pooled effect	828	100.0%	0.03 [0.02, 0.04]	7.61 (P < 0.00001)	99%
	Walking (greater flexibility)	Wolf et al. [8]	18	100.0%	0.03 [-0.01, 0.07]	1.50 (P = 0.13)	N/A

Step time (s)	Athletic	Lythgo et al. [7]*	728	100.0%	0.01 [0.01, 0.02]	5.25 (P < 0.00001)	99%

Time	Oxford	Wilkinson et al. [20]	31	100.0%	-0.40 [-1.98, 1.18]	0.50 (P = 0.62)	N/A
	Athletic	Wilkinson et al. [20]	30	100.0%	-0.20 [-1.98, 1.58]	0.22 (P = 0.83)	N/A

Cadence (steps/min)	Athletic	Lythgo et al. [7]*	471	70.5%	-5.68 [-9.05, -2.31]	-	100%

	Unknown	Moreno-Hernandez et al.[10]	61	11.0%	-3.51 [-8.51, 1.49]	-	-
	Athletic	Oeffinger et al. [9]	14	4.2%	-8.30 [-19.76, 3.16]	-	-
	Oxford	Wilkinson et al. [20]	31	4.1%	-2.10 [-13.80, 9.60]	-	-
	Walking	Wolf et al. [8]	18	10.3%	-8.70 [-14.11, -3.29]	-	-
	Combined	Pooled effect	564	100.0%	-5.71 [-8.39, -3.02]	4.16 (P < 0.0001)	99%
	Oxford	Wilkinson et al. [20]	31	100.0%	-0.20 [-9.99, 9.59	0.04 (P = 0.97)	N/A
	Walking (greater flexibility)	Wolf et al. [8]	18	100.0%	-4.60 [-9.99, 0.79]	1.67 (P = 0.09)	N/A

Support base (m)	Athletic	Lythgo et al. [7]*	753	99.1%	0.01 [0.00, 0.01]	-	89%
	Oxford	Wegener et al. [23]	20	0.5%	0.01 [-0.01, 0.03]	-	-
	Oxford	Wilkinson et al. [20]	31	0.4%	0.01 [-0.00, 0.03]	-	-
	Combined	Pooled effect	804	100.0%	0.01 [0.00, 0.01]	9.23 (P < 0.00001)	96%
	Athletic	Wilkinson et al. [20]	30	100.0%	0.00 [-0.01, 0.02]	0.49 (P = 0.62)	N/A

Toe-off (%) of gait cycle	Walking	Wolf et al. [8]	18	100.0%	2.30 [1.61, 2.99]	6.56 (P < 0.00001)	N/A

	Walking (greater flexibility)	Wolf et al. [8]	18	100.0%	2.20 [1.51, 2.89]	6.28 (P < 0.00001)	N/A

Double support (%)	Athletic	Lythgo et al.*	898	100.0%	1.53 [1.30, 1.77]	-	99%

	Oxford	Wegener et al. [23]	20	0.0%	2.49 [-14.15, 19.13]	-	-
	Combined	Pooled effect	918	100.0%	1.54 [1.27, 1.80]	11.40 (P < 0.00001)	99%

Single support (%)	Athletic	Lythgo et al. [7]*	898	100.0%	-0.79 [-0.92, -0.65]	11.26 (P < 0.00001)	99%

Stance time (%)	Athletic	Lythgo et al. [7]*	898	98.50%	0.81 [0.70, 0.92]	-	-
	Unknown	Moreno-Hernandez et al.[10]	61	1.5%	0.74 [-0.12, 1.60]	-	-
	Combined	Pooled effect	959	100.0%	0.81 [0.70, 0.92]	14.24 (P < 0.00001)	98%

Swing time (%)	Shoe	Moreno-Hernandez et al.[10]	61	100.0%	-0.74 [-1.60, 0.12]	1.68 (P = 0.09)	N/A

Contact time (ms)	Walking	Kristen et al. [15]	30	100%	49.00 [-9.88, 107.88]	1.63 (P = 0.10)	N/A

Angle of gait (°)	Athletic	Lythgo et al. [7]*	898	99.9%	-0.03 [-0.34, 0.28]	-	98%
	Walking	Wolf et al. [8]	18	0.1%	-3.10 [-16.02, 9.82]	-	-
	Combined	Pooled effect	916	100.0%	-0.03 [-0.35, 0.29]	0.19 (P = 0.85)	98%
	Walking (greater flexibility)	Wolf et al. [8]	18	100.0%	-2.50 [-5.58, 0.58]	1.59 (P = 0.11)	N/A

Progression angle (°)	Oxford	Wilkinson et al. [20]	31	100.0%	-2.50 [-7.32, 2.32]	1.02 (P = 0.31)	N/A
	Athletic	Wilkinson et al. [20]	30	100.0%	-0.40 [-5.19, 4.39]	0.16 (P = 0.87)	N/A

A negative mean difference value indicates a decrease during shod walking compared to barefoot walking. *Pooled effect calculated using inverse variance method in Review manager 5.0 for all eligible reported data. N/A indicates not applicable.

The findings for mean difference, 95% CI, statistical significance, weighting and heterogeneity of running spatio-temporal variables are presented in Table 4. Additional running spatio-temporal details, including barefoot and shod values for each study, are reported in Additional File 2. There were no differences between barefoot running and shod running.

**Table 4:** Mean differences and statistical significance for spatio-temporal variables for shod and barefoot running.

Variable	Shoe Condition	Authors	n	Weighting	Mean difference [95%CI]	Statistical significance: z Score (P)	**Heterogeneity: *I***^**2**^**%**
Running velocity (m/s)	Unknown	Lieberman et al. [25]	17	100.0%	-0.20 [-0.54, 0.14]	1.17 (P = 0.24)	N/A

Sprinting velocity (m/s)	Unknown	Tazuke [26]	4	100.0%	-0.16 [-0.77, 0.45]	0.52 (P = 0.60)	N/A

A negative mean difference value indicates a decrease during shod running compared to barefoot running. N/A indicates not applicable

### Kinematic findings

The findings for mean difference, 95% CI, statistical significance, weighting and heterogeneity of kinematic variables while walking are presented in Table 5. Additional walking kinematic details, including barefoot and shod values for each study, are reported in Additional File 3. Compared to barefoot, shod walking resulted in: increased sagittal plane tibia-rearfoot range of motion (ROM); increased tibia-foot ROM in athletic shoes; increased max-plantarflexion in athletic shoes; increased ankle ROM from foot lift to max-plantarflexion; decreased ankle max-dorsiflexion in Oxford shoes; decreased plantarflexion at foot lift in Oxford shoes; increased 'subtalar' rotation ROM; increased sagittal plane knee ROM; decreased hallux ROM; reduced change in the length of the medial arch; decreased foot torsion ROM; decreased forefoot supination ROM; decreased widening of the forefoot; decreased sagittal plane midfoot ROM; decreased frontal plane midfoot ROM; and decreased transverse plane midfoot ROM.

**Table 5:** Mean differences and statistical significance for kinematic variables for shod and barefoot walking.

Variable	Shoe Condition	Authors	n	Weighting	Mean difference [95%CI]	Statistical significance: z Score (P)	**Heterogeneity: *I***^**2**^**%**
Hallux flexion ROM(°)	Oxford	Wegener et al. [23]	20	64.5%	-11.52 [-13.64, -9.40]	-	-
	Walking	Wolf et al. [8]	18	35.5%	-11.40 [-14.26, -8.54]	-	-
	Combined	Pooled effect	38	100.0%	-11.48 [-13.18, -9.78]	13.22 (P < 0.00001)	0%
	Walking (increased flexibility)	Wolf et al. [8]	18	100.0%	-9.30 [-12.29, -6.31]	6.09 (P < 0.00001)	N/A

Sagittal tibia-rearfoot ROM (°)	Oxford	Wegener et al. [23]	20	43.5%	1.24 [-1.80, 4.28]	-	-
	Walking	Wolf et al. [8]	18	56.5%	4.10 [1.84, 6.36]	-	-
	Combined	Pooled effect	38	100.0%	2.86 [0.08, 5.64]	2.01 (P = 0.04)	54%
	Walking (increased flexibility)	Wolf et al. [8]	18	100.0%	3.20 [0.91, 5.49]	2.74 (P = 0.006)	N/A

Sagittal tibia-foot ROM (°)	Oxford	Wilkinson et al. [20]	27	49.3%	6.40 [3.40, 9.40]	-	-
	Walking	Wolf et al. [8]	18	50.4%	-0.80 [-3.53, 1.93]	-	-
	Combined	Pooled effect	45	100.0%	2.75 [-4.31, 9.80]	0.76 (P = 0.45)	91%
	Athletic	Wilkinson et al.[20]	26	100.0%	7.60 [4.13, 11.07]	4.29 (P < 0.0001)	N/A
	Walking (increased flexibility)	Wolf et al. [8]	18	100.0%	-1.00 [-3.82, 1.82]	0.70 (P = 0.49)	N/A

Medial arch length ROM (°)	Walking	Wolf et al. [8]	18	100.0%	-4.00 [-5.35, -2.65]	5.82 (P < 0.00001)	N/A
	Walking (increased flexibility)	Wolf et al. [8]	18	100.0%	-3.90 [-5.32, -2.48]	5.37 (P < 0.00001)	N/A

'Subtalar' rotation ROM(°)	Walking	Wolf et al. [8]	18	100.0%	0.90 [-0.09, 1.89]	1.78 (P = 0.07)	N/A
	Walking (increased flexibility)	Wolf et al. [8]	18	100.0%	1.10 [0.11, 2.09]	2.18 (P = 0.03)	N/A

Foot torsion ROM (°)	Walking	Wolf et al. [8]	18	100.0%	-5.10 [-6.67, -3.53]	6.36 (P < 0.00001)	N/A
	Walking (increased flexibility)	Wolf et al. [8]	18	100.0%	-4.60 [-6.27, -2.93]	5.41 (P < 0.00001)	N/A

Forefoot supination ROM (°)	Walking	Wolf et al. [8]	18	100.0%	-1.90 [-3.48, -0.32]	2.36 (P = 0.02)	N/A
	Walking (increased flexibility)	Wolf et al. [8]	18	100.0%	-1.90 [-3.40, -0.40]	2.48 (P = 0.01)	N/A

Foot rotation ROM (°)	Walking	Wolf et al. [8]	18	100.0%	-2.20 [-4.88, 0.48]	1.61 (P = 0.11)	N/A
	Walking (increased flexibility)	Wolf et al. [8]	18	100.0%	-1.50 [-4.32, 1.32]	1.04 (P = 0.30)	N/A

Forefoot width ROM (%)	Walking	Wolf et al. [8]	18	100.0%	-5.40 [-6.97, -3.83]	6.74 (P < 0.00001)	N/A
	Walking (increased flexibility)	Wolf et al. [8]	18	100.0%	-3.80 [-5.37, -2.23]	4.74 (P < 0.00001)	N/A

Midfoot sagittal plane ROM (°)	Oxford	Wegener et al.[23]	20	100.0%	-7.44 [-11.15, -3.73]	3.93 (P < 0.0001)	N/A

Midfoot frontal plane ROM (°)	Oxford	Wegener et al. [23]	20	100.0%	-3.07 [-5.04, -1.10]	3.06 (P = 0.002)	N/A

Midfoot transverse plane ROM (°)	Oxford	Wegener et al. [23]	20	100.0%	-5.01 [-6.55, -3.48]	6.39 (P < 0.00001)	N/A

Rearfoot frontal plane ROM (°)	Oxford	Wegener et al. [23]	20	100.0%	-1.68 [-4.27, 0.90]	1.28 (P = 0.20)	N/A

Rearfoot transverse plane ROM (°)	Oxford	Wegener et al. [23]	20	100.0%	0.39 [-2.52, 3.29]	0.26 (P = 0.79)	N/A

Knee sagittal plane ROM (°)	Oxford	Wegener et al. [23]	20	100.0%	9.21 [3.22, 15.21]	3.01 (P = 0.003)	N/A

Knee frontal plane ROM (°)	Oxford	Wegener et al. [23]	20	100.0%	0.02 [-1.48, 1.52]	0.02 (P = 0.98)	N/A

Knee transverse plane ROM (°)	Oxford	Wegener et al. [23]	20	100.0%	-0.13 [-4.80, 4.55]	0.05 (P = 0.96)	N/A

Hip sagittal plane ROM (°)	Oxford	Wegener et al. [23]	20	100.0%	2.04 [-1.21, 5.29]	1.23 (P = 0.22)	N/A

Hip frontal plane ROM (°)	Oxford	Wegener et al. [23]	20	100.0%	-0.40 [-2.39, 1.58]	0.40 (P = 0.69)	N/A

Hip transverse plane ROM (°)	Oxford	Wegener et al. [23]	20	100.0%	1.10 [-1.05, 3.25]	1.00 (P = 0.32)	N/A

Ankle max dorsiflexion (°)	Oxford	Wilkinson et al.[20]	27	100.0%	-7.20 [-11.18, -3.22]	3.54 (P = 0.0004)	N/A
	Athletic	Wilkinson et al.[20]	26	100.0%	-1.70 [-5.45, 2.05]	0.89 (P = 0.37)	N/A

Ankle angle at foot lift (°)	Oxford	Wilkinson et al.[20]	27	100.0%	-5.70 [-10.45, -0.95]	2.35 (P = 0.02)	N/A
	Athletic	Wilkinson et al.[20]	26	100.0%	-1.50 [-5.92, 2.92]	0.67 (P = 0.51)	N/A

Ankle max plantarflexion (°)	Oxford	Wilkinson et al.[20]	27	100.0%	-0.70 [-5.94, 4.54]	0.26 (P = 0.79)	N/A
	Athletic	Wilkinson et al.[20]	26	100.0%	5.80 [1.58, 10.02]	2.69 (P = 0.007)	N/A

Ankle ROM, foot lift to max plantarflexion (°)	Oxford	Wilkinson et al.[20]	27	100.0%	5.00 [1.79, 8.21]	3.05 (P = 0.002)	N/A
	Athletic	Wilkinson et al.[20]	26	100.0%	7.30 [3.56, 11.04]	3.82 (P = 0.0001)	N/A

A negative mean difference value indicates a decrease during shod walking compared to barefoot walking. N/A indicates not applicable.

The mean difference, 95% CI, statistical significance, weighting and heterogeneity of kinematic range of motion variables while running are presented in Table 6. Additional running kinematic details, including barefoot and shod values for each study, are reported in Additional File 4. Compared to barefoot running, significant changes during shod running were: reduced ankle plantarflexion angle at foot strike; reduced plantar foot angle at foot strike (angle between the ground and the plantar surface of the foot/shoe); decreased angular velocity of the knee; and decreased swing-back velocity of the tibia. Lieberman and co-investigators, [25] reported that rearfoot strike mode increased from 62% to 97% during shod running while midfoot and forefoot strike reduced from 19% for both to 3% and 0% respectively.

**Table 6:** Mean differences and statistical significance for kinematic variables for shod and barefoot running.

Variable	Shoe Condition	Authors	n	Weighting	Mean difference [95%CI]	Statistical significance: z Score (P)	Heterogeneity: *I*^2^%
Ankle angle at foot strike (°)	Unknown	Lieberman et al. [25]	17	100.0%	-6.80 [-13.52, -0.08]	1.98 (P = 0.049)	N/A

Plantar foot angle at foot strike (°)	Unknown	Lieberman et al. [25]	17	100.0%	-9.70 [-16.43, -2.97]	2.83 (P = 0.005)	N/A

Knee angle at foot strike (°)	Unknown	Lieberman et al. [25]	17	100.0%	-0.50 [-4.90, 3.90]	0.22 (P = 0.82)	N/A

Knee lift angle (°)	Unknown	Tazuke [26]	4	100.0%	-1.20 [-16.25, 13.84]	0.16 (P = 0.88)	N/A

Knee angular velocity (°/s)	Unknown	Tazuke [26]	4	100.0%	-160.59 [-304.34, -16.83]	2.19 (P = 0.03)	N/A

Swing-back velocity (°/s)	Unknown	Tazuke [26]	4	100.0%	-84.24 [-158.64, -9.84]	2.22 (P = 0.03)	N/A

A negative mean difference value indicates a decrease during shod running compared to barefoot running. N/A indicates not applicable

### Kinetic findings

The mean difference, 95% CI, statistical significance, weighting and heterogeneity of kinetic variables during walking are presented in Table 7. Additional walking kinetic details, including barefoot and shod values for each study, are reported in Additional File 5. No significant differences were found in kinetic walking variables. However, a higher vertical ground reaction force for shod walking was reported by Kristen and co-researchers [15] using the less cautious Chi-Square test for significance.

**Table 7:** Mean differences and statistical significance for kinetic variables for shod and barefoot walking.

Variable	Shoe Condition	Authors	n	Weighting	Mean difference [95%CI]	Statistical significance: z Score(P)	**Heterogeneity: *I***^**2**^**%**
Vertical ground reaction force (%BW)	Walking	Kristen et al. [15]	30	100.0%	6.30 [-2.82, 15.42]	1.35 (P = 0.18)	N/A

Anterior Posterior Max GRF (%BW)	Walking	Kristen et al. [15]	30	100.0%	-0.90 [-3.66, 1.86]	0.64 (P = 0.52)	N/A

Anterior Posterior Min GRF (%BW)	Walking	Kristen et al. [15]	30	100.0%	-1.00 [-5.99, 3.99]	0.39 (P = 0.69)	N/A

A negative mean difference value indicates a decrease during shod walking compared to barefoot walking. N/A indicates not applicable

The mean difference, 95% CI, statistical significance, weighting and heterogeneity of kinetic variables during running are presented in Table 8. Additional running kinetic details, including barefoot and shod values for each study, are reported in Additional File 6. Compared to barefoot running, significant kinetic changes during shod running were: reduced 'long axis' maximum tibial acceleration; decreased rate of tibial acceleration; and decreased shock wave transmission as a ratio of maximum tibial acceleration. However, Alcantara and colleagues [21] using a multifactor analysis of variance (ANOVA) to test for significance, reported that vertical ground reaction force was lower in walking shoes than either athletic shoes or when barefoot for boys and girls. Boys had higher forces in athletic shoes compared to barefoot and walking shoes, where as girls had higher values unshod compared to athletic shoes and walking shoes, rate of load at impact was significantly higher during barefoot running than both shod running conditions for boys and girls [21].

**Table 8:** Mean differences and statistical significance for kinetic variables for shod and barefoot running.

Variable	Shoe Condition	Authors	n	Weighting	Mean difference [95%CI]	Statistical significance: z Score (P)	Heterogeneity: *I*^2^%
Max vertical impact force (BW)	Athletic	Alcantara et al. [21] (girls)	4	49.4%	-0.32 [-0.42, -0.22]	-	-
	Athletic	Alcantara et al. [21] (boys)	4	50.6%	0.05 [-0.01, 0.11]	-	-
	Athletic	Pooled effect	8	100.0%	-0.13 [-0.50, 0.23]	0.72 (P = 0.47)	97%
	Walking	Alcantara et al. [21] (girls)	4	49.9%	-0.16 [-0.22, -0.10]	-	-
	Walking	Alcantara et al. [21] (boys)	4	50.1%	-0.68 [-0.73, -0.63]	-	-
	Walking	Pooled effect	8	100.0%	-0.42 [-0.93, 0.09]	1.62 (P = 0.11)	99%

Rate of load at impact (BW/s)	Athletic	Alcantara et al. [21] (girls)	4	49.5%	-139.71 [-161.60, -117.82]	-	-
	Athletic	Alcantara et al. [21] (boys)	4	50.5%	-43.64 [-56.16, -31.12]	-	-
	Athletic	Pooled effect	8	100.0%	-91.24 [-185.38, 2.90]	1.90 (P = 0.06)	98%
	Walking	Alcantara et al. [21] (girls)	4	49.6%	-146.63 [-168.67, -124.59]	-	-
	Walking	Alcantara et al. [21] (boys)	4	50.4%	-41.88 [-54.47, -29.29]	-	-
	Walking	Pooled effect	8	100.0%	-93.85 [-196.50, 8.80]	1.79 (P = 0.07)	98%

Long axis max tibial acceleration (g)	Athletic	Alcantara et al. [21] (girls)	4	49.9%	-2.16 [-2.61, -1.71]	-	-
	Athletic	Alcantara et al. [21] (boys)	4	50.1%	-0.94 [-1.37, -0.51]	-	-
	Athletic	Pooled effect	8	100.0%	-1.55 [-2.74, -0.35]	2.54 (P = 0.01)	93%
	Walking	Alcantara et al. [21] (girls)	4	49.7%	-2.65 [-3.12, -2.18]	-	-
	Walking	Alcantara et al. [21] (boys)	4	50.3%	-1.67 [-2.11, -1.23]	-	-
	Walking	Pooled effect	8	100.0%	-2.16 [-3.12, -1.20]	4.40 (P < 0.0001)	89%

Rate of tibia acceleration (g/s)	Athletic	Alcantara et al. [21] (girls)	4	50.6%	-252.59 [-292.21, -212.97]	-	-
	Athletic	Alcantara et al. [21] (boys)	4	49.4%	-135.17 [-181.84, -88.50]	-	-
	Athletic	Pooled effect	8	100.0%	-194.56 [-309.62, -79.49]	3.31 (P = 0.0009)	93%
	Walking	Alcantara et al. [21] (girls)	4	56.4%	-261.63 [-302.88, -220.38]	-	-
	Walking	Alcantara et al. [21] (boys)	4	43.6%	-145.83 [-192.73, -98.93]	-	-
	Walking	Pooled effect	8	100.0%	-211.13 [-242.11, -180.16]	13.36 (P < 0.00001)	92%

Shock wave transmission as a ratio of maximum acceleration (g/BW)	Athletic	Alcantara et al. [21] (girls)	4	54.8%	-0.35 [-0.57, -0.13]	-	-
	Athletic	Alcantara et al. [21] (boys)	4	45.2%	-0.59 [-0.86, -0.32]	-	-
	Athletic	Pooled effect	8	100.0%	-0.46 [-0.69, -0.22]	3.84 (P = 0.0001)	45%
	Walking	Alcantara et al. [21] (girls)	4	50.1%	-0.14 [-0.40, 0.12]	-	-
	Walking	Alcantara et al. [21] (boys)	4	49.9%	-0.78 [-1.05, -0.51]	-	-
	Walking	Pooled effect	8	100.0%	-0.46 [-1.09, 0.17]	1.43 (P = 0.15)	91%

A negative mean difference value indicates a decrease during shod running compared to barefoot running.

### Electromyography

Mueller and co-investigators [22] reported that EMG amplitude of the tibialis anterior during weight acceptance and midstance was significantly (P < 0.05) greater during shod walking (mean 1.78) than barefoot walking (mean 1.63) using a univariate ANOVA. There were no differences for the peroneus longus, and medial gastrocnemius [22]. No additional data were able to be obtained for further meta-analysis.

## Discussion

This systematic review identified 11 studies evaluating biomechanical differences between barefoot and shod gait in children. A total of 62 variables describing barefoot and shod walking and running were examined. The maximum number of studies that were able to be combined for meta-analyses was limited to five studies between the three variables of stride length, walking velocity and cadence.

### Walking

Children walked faster when wearing shoes. Since walking cadence was found to decrease, the increase in stride length is particularly noteworthy. Possible explanations for the longer stride in shoes include that of an effective increase of leg length of approximately 1 cm to 2 cm. Indeed, in children aged between 5 and 6, a 7 cm increase in stride length can be expected for a 4 cm increase in leg length [7]. The increased stride length could also be due to the increase in mass of the shod foot, which results in increased inertia of the leg during the swing phase [9]. It is also possible that the shoe provides a perception of protection, giving confidence to the wearer to 'stride out'.

Increased double-limb support time and base of support during shod walking might be indicative of modifications to the gait pattern to improve stability [27,28]. Shoes could act as a sensory filter by reducing proprioceptive feedback, and leading to gait modifications to improve stability [29]. The increased sole width of shoes, compared to when barefoot, could also cause a child to increase their base of support to avoid contact between feet. Alternatively the greater shoe ground contact area compared to barefoot could result in the measurement of an increase in the base of support. While the increase of base of support was statistically significant, the 1 cm increase of the distance between their feet during walking may not be functionally significant. The increased time spent in double support may be due to the increased length and breadth of the shod foot which in turn would lead to longer ground contact time and delayed toe-off time during the gait cycle.

Spatio-temporal walking variables showed greater homogeneity than studies investigating other categories of biomechanical variables. Between two and five studies were able to be combined for meta-analyses for 9 of the 17 spatio-temporal walking variables.

Shoes decrease the intrinsic motion of the foot during walking. Eight of the nine range of motion variables measuring foot motion were reduced in shoes. 'Subtalar' rotation was the only range of motion variable to increase in one shoe condition, designed to have greater flexibility, possibly because of the lateral lever arm effect of footwear increasing 'subtalar' joint motion [30]. The extent of the reduced foot motion indicates that shoes have a splinting effect on foot joints. A consequence of motion reduction could be that of less stimulus to foot musculature and therefore muscle strength, since shoes with increased flexibility have been shown to increase foot muscle strength in adults [31].

The reduction of hallux motion that occurs while walking in shoes may adversely affect the 'windlass' mechanism in which winding of the plantar aponeurosis around the metatarsophalangeal joint during hallux extension assists raising the medial longitudinal arch and inverting the rearfoot following heel rise [32]. It is likely that the increases in sagittal plane motion at the ankle and knee are due to the increased stride length while walking in shoes [8,23]. Unfortunately, meta-analysis of kinematic variables was restricted by inconsistencies in biomechanical models and under-reporting of standard deviations/error. Meta-analysis of kinematic variables could only be performed for hallux ROM, tibia-rearfoot ROM and tibia-foot ROM between two studies [8,20,23].

### Running

Vertical ground reaction force does not seem to be reduced by shoes during running. This interesting finding concurs with adult footwear research showing that forces are relatively unchanged during barefoot and shod running [33]. However, shoes appear to attenuate loading since long-axis tibial acceleration was reduced during shod running in children. In addition, there was a trend for the rate of load at impact to be reduced by shoes.

Sprinting with shoes resulted in decreased angular velocity of the knee joint and swing back velocity of the tibia [26]. The increased weight of shoes on the end of the foot and the consequent increase in the moment of inertia may be responsible for these changes.

During shod running there was an increase in the prevalence of a rearfoot strike pattern from 62% barefoot to 97% shod [25]. There was a corresponding decrease of forefoot and midfoot strike patterns [25]. This change in pattern from barefoot to shod running is a consistent finding with that of adults [25,33]. It has previously been hypothesised that a forefoot and midfoot strike pattern while running barefoot is a strategy to improve shock attenuation [25,33]. Interestingly, the majority of children (62%) ran with a rearfoot strike pattern whilst barefoot [25].

### Quality assessment

The majority of the included studies had moderate methodological quality. The main limitations were with external and internal validity, selection and confounding biases. Although blinding and randomisation are considered to have the greatest confounding effects [13], only five studies randomised the order of assessment [9,20-23] and no study blinded the participants to shoe interventions. While blinding is difficult to achieve with barefoot gait, randomisation of assessment should be implemented in future studies to improve methodological quality. While there was a potential for bias in this review by including non-randomised studies, the effect of carryover between interventions in repeated measures studies was considered small compared to the chance of a type I error by not including these studies.

### Clinical implications

In this systematic review, 45 of the 62 (73%) biomechanical comparisons between barefoot and shod gait were statistically significant. Shoes therefore have a substantial effect on the gait of children. The extent of the biomechanical differences between barefoot and shod gait warrants further investigation into the effects of shoes on long-term growth and development of children. While the review included participants aged 1.6 to 15 years all but 3 studies included children in middle childhood (7-11 years), meaning extrapolation of the results of the review to children outside this age range should be done with some caution. The clinical assessment of shoe and in-shoe interventions in children should consider the numerous effects of shoes on their gait. Perhaps a standardised shod condition could be utilised during the clinical assessment and prescription of in-shoe interventions to ensure that any improvement is due to the intervention, rather than the shoe only.

From this review it is not possible to prescribe the optimal shoe for children. Nonetheless, previous reviews have suggested that children's shoes should be based on the barefoot model [11]. However, since the design of some of the shoes examined in the current review were designed on these recommendations and still result in considerable differences between barefoot and shod walking [8], further refinement to children's shoes in respect to foot function, proprioception and stability is required. Future research could investigate the effects of specific shoe modifications on proprioception and the walking and running gait of children. Further attention could also be paid to reducing the weight of shoes which may be responsible for some of the changes found in children's walking and running gait.

The findings of this review will help guide future research, including the investigation of the long-term impacts of the differences between barefoot and shod gait on paediatric growth and development. While diversity in methodology is the nature of biomechanics research, inconsistencies of variables investigated by different study groups restricted the pooling of data and the ability to draw clear conclusions. A universal set of recommendations for reporting the most valid and reliable gait parameters might assist the evaluation of the iatrogenic or the therapeutic effects of shoes. These variables should closely reflect events or movements in the gait cycle and avoid the creation of abstract composite variables with reduced clinical or functional relevance. A shift in reporting practices in the biomechanics literature to report change scores and their corresponding variability would assist future statistical meta-analysis by allowing the use of less conservative statistical tests such as the generic inverse variance method, thereby reducing the risk of type 1 error [13].

## Conclusion

Shoes affect the gait of children. With shoes, children walk faster by taking longer steps with greater ankle and knee motion and increased tibialis anterior activity. Shoes reduce foot motion and increase the support phases of the gait cycle. During running, shoes reduce swing phase leg speed, attenuate some shock and encourage a rearfoot strike pattern. The impact of footwear on gait should be considered when assessing the paediatric patient and evaluating the effect of shoe or in-shoe interventions.

## Competing interests

The authors declare that they have no competing interests.

## Authors' contributions

CW led and designed the review, carried out searches, eligibility checks, performed quality assessment, extracted data, performed meta-analysis, interpreted the findings and drafted the manuscript. AEH assisted in designing the review, carried out eligibility checks, performed quality assessment, checked extracted data, assisted in the interpretation of the findings and the drafting of the manuscript. BV assisted in designing the review, performed quality assessment of studies published in German, assisted in the interpretation of the findings and in the drafting of the manuscript. JB assisted in designing the review methodology, interpretation of the findings and in the drafting of the manuscript. RMS assisted in the interpretation of the findings and in the drafting of the manuscript. All authors read and approved the final manuscript.

## Supplementary Material

Additional file 1 **Spatio-temporal variables for barefoot and shod walking**. Click here for file

Additional file 2 **Spatio-temporal variables for barefoot and shod running**. Click here for file

Additional file 3 **Kinematic variables for barefoot and shod walking**. Click here for file

Additional file 4 **Kinematic variables for barefoot and shod running**. Click here for file

Additional file 5 **Kinetic variables for barefoot and shod walking**. Click here for file

Additional file 6 **Kinetic variables for barefoot and shod running**. Click here for file
