# Supplementary material for: Effect of children's shoes on gait: a systematic review and meta-analysis
Source: J Foot Ankle Res. 2011 Jan 18;4:3. doi: 10.1186/1757-1146-4-3 (PMC3031211; doi:10.1186/1757-1146-4-3)
Supplement: Additional file 1 — Spatio-temporal variables for barefoot and shod walking. [file 1757-1146-4-3-S1.DOC]

**Additional File 1:** Mean differences and statistical significance for spatio-temporal variables for shod and barefoot walking.

| **Variable** | **Shoe Condition** | **Authors** | **n** | **Shod: mean(SD)** | **Barefoot:**  **mean(SD)** | **Mean difference [95%CI]** | **Weighting** | **Statistical significance: z Score (P)** | **Heterogeneity: *I*^2^%** |
| --- | --- | --- | --- | --- | --- | --- | --- | --- | --- |
| Velocity (m/s) | Sports | Lythgo et al. [7] 5 yrs | 69 | 1.30 (0.016) | 1.25(0.018) | 0.05 [0.05, 0.06] | 11.7% | - | - |
|  | Sports | Lythgo et al. [7] 6 yrs | 140 | 1.34 (0.024) | 1.28(0.022) | 0.06 [0.05, 0.07] | 11.8% | - | - |
|  | Sports | Lythgo et al. [7] 7 yrs | 152 | 1.35 (0.016) | 1.26(0.015) | 0.09 [0.08, 0.09] | 12.0% | - | - |
|  | Sports | Lythgo et al. [7] 8 yrs | 145 | 1.39 (0.021) | 1.31(0.023) | 0.09 [0.08, 0.09] | 11.8% | - | - |
|  | Sports | Lythgo et al. [7] 9 yrs | 117 | 1.35 (0.016) | 1.27(0.015) | 0.08 [0.08, 0.09] | 11.9% | - | - |
|  | Sports | Lythgo et al. [7] 10 yrs | 108 | 1.40 (0.014) | 1.30(0.012) | 0.10 [0.09, 0.10] | 12.0% | - | - |
|  | Sports | Lythgo et al. [7] 11 yrs | 105 | 1.40 (0.017) | 1.32(0.015) | 0.08 [0.07, 0.08] | 11.9% | - | - |
|  | Sports | Lythgo et al. [7] 12 yrs | 62 | 1.39 (0.015) | 1.34(0.014) | 0.05 [0.05, 0.06] | 11.8% | - | - |
|  | Unknown | Moreno-Hernandez et al.[10] | 61 | 1.19(0.18) | 1.13 (0.20) | 0.05 [-0.01, 0.12] | 2.2% | - | - |
|  | Sports | Oeffinger et al. [9] | 14 | 1.43 (0.15) | 1.39 (0.17) | 0.04 [-0.08, 0.16] | 0.8% | - | - |
|  | Oxford | Wegener et al.[23] | 20 | 1.35 (0.19) | 1.32 (0.18) | 0.03 [-0.08, 0.14] | 0.9% | - | - |
|  | Walking | Wolf et al. [8] | 18 | 1.28 (0.13) | 1.29 (0.14) | -0.01 [-0.10, 0.08] | 1.4% | - | - |
|  | Combined | Pooled effect | 1011 | - | - | 0.07 [0.06, 0.08] | 100.0% | 12.97 (P < 0.00001) | 97% |
|  | Walking (greater flexibility) | Wolf et al. [8] | 18 | 1.31 (0.15) | 1.29 (0.14) | 0.02 [-0.07, 0.11] | 100.0% | 0.41 (P = 0.68) | N/A |
| Stride length (m) | Sports | Lythgo et al. [7] 5 yrs | 69 | 1.10 (0.009) | 0.97 (0.009) | 0.12 [0.12, 0.13] | 14.2% | - | - |
|  | Sports | Lythgo et al. [7] 6 yrs | 140 | 1.157 (0.012) | 1.05 (0.01) | 0.11 [0.11, 0.11] | 14.3% | - | **-** |
|  | Sports | Lythgo et al. [7] 7yrs | 152 | 1.203 (0.010) | 1.09 (0.01) | 0.11 [0.11, 0.11] | 14.5% | - | **-** |
|  | Sports | Lythgo et al. [7] 8 yrs | 145 | 1.26 (0.010) | 1.15 (0.009) | 0.11 [0.11, 0.11] | 14.5% | - | **-** |
|  | Sports | Lythgo et al. [7] 10 yrs | 108 | 1.344 (0.012) | 1.23 (0.01) | 0.12 [0.11, 0.12] | 14.1% | - | - |
|  | Sports | Lythgo et al. [7] 11 yrs | 105 | 1.374 (0.012) | 1.27 (0.01) | 0.10 [0.10, 0.11] | 14.1% | - | **-** |
|  | Sports | Lythgo et al. [7] 12 yrs | 62 | 1.44 (0.021) | 1.34 (0.02) | 0.09 [0.09, 0.10] | 11.9% | - | **-** |
|  | Unknown | Moreno-Hernandez et al.[10] | 61 | 1.21 (0.14) | 1.13 (0.14) | 0.07 [0.02, 0.12] | 1.10% | - | **-** |
|  | Sports | Oeffinger et al. [9] | 12 | 1.37 (0.12) | 1.25 (0.14) | 0.12 [0.02, 0.21] | 0.30% | - | **-** |
|  | Oxford | Wegener et al. [23] | 20 | 1.34 (0.19) | 1.23 (0.15) | 0.11 [0.00, 0.22] | 0.20% | - | **-** |
|  | Walking | Wolf et al. [8] | 18 | 1.24 (0.09) | 1.17 (0.1) | 0.07 [0.01, 0.13] | 0.70% | - | **-** |
|  | Combined | Pooled effect | 894 | - | - | 0.11 [0.10, 0.12] | 100.0% | 40.49 (P < 0.00001) | 93% |
|  | Walking (greater flexibility) | Wolf et al. [8] | 18 | 1.23 (0.11) | 1.17 (0.1) | 0.06 [-0.01, 0.13] | 100.0% | 1.71 (P = 0.09) | N/A |
| Step length (%) | Walking | Kristen et al. [15] | 30 | 100.2 (4.7) | 100.0 (5.0) | 0.20 [-2.26, 2.66] | 6.2% | **-** | **-** |
|  | Sports | Lythgo et al. [7] 5 yrs | 69 | 112.53 (0.34) | 100.0 (0.27) | 12.53 [12.42, 12.63] | 12.5% | **-** | **-** |
|  | Sports | Lythgo et al. [7] 6 yrs | 140 | 110.73 (0.27) | 100.0 (0.24) | 10.73 [10.67, 10.79] | 12.5% | **-** | **-** |
|  | Sports | Lythgo et al. [7] 7 yrs | 152 | 109.49 (0.31) | 100.0 (0.28) | 9.49 [9.42, 9.56] | 12.5% | **-** | **-** |
|  | Sports | Lythgo et al. [7] 8 yrs | 145 | 110.12 (0.29) | 100.0 (0.26) | 10.12 [10.06, 10.19] | 12.5% | **-** | **-** |
|  | Sports | Lythgo et al. [7] 10 yrs | 108 | 109.62 (0.41) | 100.0 (0.38) | 9.62 [9.52, 9.73] | 12.5% | **-** | **-** |
|  | Sports | Lythgo et al. [7] 11 yrs | 105 | 108.02 (0.42) | 100.0 (0.36) | 8.02 [7.91, 8.12] | 12.5% | **-** | **-** |
|  | Sports | Lythgo et al. [7] 12 yrs | 62 | 107.31 (0.77) | 100.0 (0.62) | 7.31 [7.07, 7.56] | 12.4% | **-** | **-** |
|  | Unknown | Moreno-Hernandez et al.[10] | 61 | 106.57 (6.92) | 100.0 (6.74) | 6.57 [4.14, 8.99] | 6.3% | - | - |
|  | Combined | Pooled effect | 872 | - | - | 8.90 [8.04, 9.77] | 100.0% | 20.16 (P < 0.00001) | 100% |
| Length (m) | Oxford | Wilkinson et al. [20] | 31 | 0.409 (0.089) | 0.379 (  0.064) | 0.03 [-0.01, 0.07] | 100.0% | 1.52 (P=0.13) | N/A |
|  | Athletic | Wilkinson et al. [20] | 30 | 0.415 (0.061) | 0.379 (  0.064) | 0.04 [0.00, 0.07] | 100.0% | 2.25 (P=0.02) | N/A |
| Stride time (s) | Sports | Lythgo et al. [7] 5 yrs | 69 | 0.85 (0.01) | 0.80 (0.01) | 0.05 [0.05, 0.05] | 13.2% | **-** | **-** |
|  | Sports | Lythgo et al. [7] 6 yrs | 140 | 0.88 (0.007) | 0.83(0.007) | 0.05 [0.04, 0.05] | 13.5% | **-** | **-** |
|  | Sports | Lythgo et al. [7] 7 yrs | 152 | 0.90 (0.007) | 0.88 (0.007) | 0.02 [0.02, 0.03] | 13.5% | **-** | **-** |
|  | Sports | Lythgo et al. [7] 8 yrs | 145 | 0.91 (0.008) | 0.89 (0.007) | 0.02 [0.02, 0.03] | 13.5% | **-** | **-** |
|  | Sports | Lythgo et al. [7] 9 yrs | 117 | 0.95 (0.007) | 0.94 (0.008) | 0.02 [0.02, 0.02] | 13.5% | **-** | **-** |
|  | Sports | Lythgo et al. [7] 11 yrs | 105 | 0.99 (0.007) | 0.97 (0.007) | 0.02 [0.02, 0.02] | 13.5% | **-** | **-** |
|  | Sports | Lythgo et al. [7] 12 yrs | 62 | 1.04 (0.01) | 1.01 (0.009) | 0.03 [0.03, 0.03] | 13.3% | **-** | **-** |
|  | Oxford | Wegener et al. [23] | 20 | 0.99 (0.08) | 0.91(0.07) | 0.08 [0.03, 0.13] | 2.6% | **-** | **-** |
|  | Walking | Wolf et al. [8] | 18 | 0.98 (0.06) | 0.91(0.06) | 0.07 [0.03, 0.11] | 3.4% | **-** | **-** |
|  | Combined | Pooled effect | 828 | - | - | 0.03 [0.02, 0.04] | 100.0% | 7.61 (P < 0.00001) | 99% |
|  | Walking (greater flexibility) | Wolf et al. [8] | 18 | 0.94 (0.06) | 0.91(0.06) | 0.03 [-0.01, 0.07] | 100.0% | 1.50 (P = 0.13) | N/A |
| Step time (s) | Sports | Lythgo et al. [7] 5 yrs | 69 | 0.85 (0.01) | 0.8 (0.01) | 0.05 [0.04, 0.05] | 14.1% | **-** | **-** |
|  | Sports | Lythgo et al. [7] 6 yrs | 140 | 0.88 (0.007) | 0.83(0.008) | 0.05 [0.04, 0.05] | 14.4% | **-** | **-** |
|  | Sports | Lythgo et al. [7] 7 yrs | 152 | 0.90 (0.008) | 0.88 (0.007) | 0.02 [0.02, 0.02] | 14.4% | **-** | **-** |
|  | Sports | Lythgo et al. [7] 8 yrs | 145 | 0.91 (0.007) | 0.89 (0.007) | 0.02 [0.02, 0.02] | 14.4% | **-** | **-** |
|  | Sports | Lythgo et al. [7] 9 yrs | 117 | 0.95(0.007) | 0.94 (0.007) | 0.01 [0.01, 0.01] | 14.3% | **-** | **-** |
|  | Sports | Lythgo et al. [7] 11 yrs | 105 | 0.99 (0.007) | 0.97(0.007) | 0.02 [0.02, 0.02] | 14.3% | **-** | **-** |
|  | Sports | Lythgo et al. [7] 12 yrs | 62 | 1.04 (0.01) | 1.01(0.009) | 0.03 [0.03, 0.03] | 14.1% | **-** | **-** |
| Time | Oxford | Wilkinson et al. [20] | 31 | 30.2 (3.2) | 30.6 (2.7) | -0.40 [-1.98, 1.18] | 100.0% | 0.50 (P=0.62) | N/A |
|  | Athletic | Wilkinson et al. [20] | 30 | 30.4 (3.8) | 30.6 (2.7) | -0.20 [-1.98, 1.58] | 100.0% | 0.22 (P=0.83) | N/A |
|  | Combined | Pooled effect | 728 | - | - | 0.01 [0.01, 0.02] | 100.0% | 5.25 (P < 0.00001) | 99% |
| Cadence (steps/min) | Sports | Lythgo et al. [7] 5 yrs | 69 | 142.8 (1.74) | 152.6(2.09) | -9.80 [-10.44, -9.16] | 17.5% | **-** | **-** |
|  | Sports | Lythgo et al. [7] 6 yrs | 140 | 138.4(1.07) | 146.3 (1.33) | -7.90 [-8.18, -7.62] | 17.6% | **-** | **-** |
|  | Sports | Lythgo et al. [7] 8 yrs | 145 | 132.8 (1.02) | 136.1(1.02) | -3.30 [-3.53, -3.07] | 17.7% | **-** | **-** |
|  | Sports | Lythgo et al. [7] 9 yrs | 117 | 127.4 (0.97) | 129.1(0.97) | -1.70 [-1.95, -1.45] | 17.7% | **-** | **-** |
|  | Shoe | Moreno-Hernandez et al.[10] | 61 | 118.97 (14.35) | 122.48(13.83) | -3.51 [-8.51, 1.49] | 11.0% | **-** | **-** |
|  | Sports | Oeffinger et al. [9] | 14 | 126.0 (13.52) | 134.3(16.29) | -8.30 [-19.76, 3.16] | 4.2% | **-** | **-** |
|  | Oxford | Wilkinson et al. [20] | 31 | 155.5 (27.8) | 157.6 (18.2) | -2.10 [-13.80, 9.60] | 4.1% | **-** | **-** |
|  | Walking | Wolf et al. [8] | 18 | 123.5(7.6) | 132.2(8.9) | -8.70 [-14.11, -3.29] | 10.3% | **-** | **-** |
|  | Combined | Pooled effect | 595 | - | - | -5.71 [-8.39, -3.02] | 100.0% | 4.16 (P < 0.0001) | 99% |
|  | Walking (greater flexibility) | Wolf et al. [8] | 18 | 127.6 (7.56) | 132.2 (8.9) | -4.60 [-9.99, 0.79] | 100.0% | 1.67 (P = 0.09) | N/A |
| Support base (m) | Sports | Lythgo et al. [7] 5 yrs | 69 | 0.077 (0.002) | 0.071 (0.002) | 0.01 [0.01, 0.01] | 14.0% | **-** | **-** |
|  | Sports | Lythgo et al. [7] 6 yrs | 140 | 0.077 (0.002) | 0.071 (0.002) | 0.01 [0.01, 0.01] | 14.5% | **-** | **-** |
|  | Sports | Lythgo et al. [7] 7 yrs | 152 | 0.081(0.002) | 0.075 (0.002) | 0.01 [0.01, 0.01] | 14.5% | **-** | **-** |
|  | Sports | Lythgo et al. [7] 9 yrs | 117 | 0.088 (0.002) | 0.083 (0.002) | 0.00 [0.00, 0.01] | 14.5% | **-** | **-** |
|  | Sports | Lythgo et al. [7] 10 yrs | 108 | 0.089 (0.002) | 0.082 (0.002) | 0.01 [0.01, 0.01] | 14.4% | **-** | **-** |
|  | Sports | Lythgo et al. [7] 11 yrs | 105 | 0.086 (0.003) | 0.084 (0.002) | 0.00 [0.00, 0.00] | 14.0% | **-** | **-** |
|  | Sports | Lythgo et al. [7] 12 yrs | 62 | 0.085 (0.003) | 0.082 (0.003) | 0.00 [0.00, 0.00] | 13.2% | **-** | **-** |
|  | Oxford | Wegener et al. [23] | 20 | 0.1 (0.02) | 0.09 (0.03) | 0.01 [-0.01, 0.03] | 0.5% | **-** |  |
|  | Oxford | Wilkinson et al. [20] | 31 | 0.103 (0.038) | 0.089 (0.03) | 0.01 [-0.00, 0.03] | 0.4% | **-** | **-** |
|  | Combined | Pooled effect | 773 | - | - | 0.01 [0.00, 0.01] | 100.0% | 9.15 (P < 0.00001) | 96% |
|  | Athletic | Wilkinson et al. [20] | 30 | 0.093 (0.033) | 0.089 (0.03) | 0.00 [-0.01, 0.02] | 100.0% | 0.49 (P=0.62) | N/A |
| Toe-off (%) of gait cycle | Walking | Wolf et al. [8] | 18 | 64.0 (1.1) | 61.7 (1.0) | 2.30 [1.61, 2.99] | 100.0% | 6.56 (P < 0.00001) | N/A |
|  | Walking (greater flexibility) | Wolf et al. [8] | 18 | 63.9 (1.1) | 61.7 (1.0) | 2.20 [1.51, 2.89] | 100.0% | 6.28 (P < 0.00001) | N/A |
| Double support (%) | Sports | Lythgo et al. [7] 5 yrs | 69 | 14.6 (0.36) | 12.2 (0.31) | 2.40 [2.29, 2.51] | 12.4% | **-** | **-** |
|  | Sports | Lythgo et al. [7] 6 yrs | 140 | 13.4 (0.46) | 11.8 (0.31) | 1.60 [1.51, 1.69] | 12.5% | **-** | **-** |
|  | Sports | Lythgo et al. [7] 7 yrs | 152 | 13.5 (0.31) | 12.4 (0.26) | 1.10 [1.04, 1.16] | 12.6% | **-** | **-** |
|  | Sports | Lythgo et al. [7] 8 yrs | 145 | 13.8 (0.26) | 12.4 (0.20) | 1.40 [1.35, 1.45] | 12.6% | **-** | **-** |
|  | Sports | Lythgo et al. [7] 9 yrs | 117 | 14.2 (0.36) | 12.8 (0.26) | 1.40 [1.32, 1.48] | 12.5% | **-** | **-** |
|  | Sports | Lythgo et al. [7] 10 yrs | 108 | 13.9 (0.31) | 12.9 (0.20) | 1.00 [0.93, 1.07] | 12.6% | **-** | **-** |
|  | Sports | Lythgo et al. [7] 11 yrs | 105 | 15.4(0.41) | 13.9 (0.36) | 1.50 [1.40, 1.60] | 12.4% | **-** | **-** |
|  | Sports | Lythgo et al. [7] 12 yrs | 62 | 15.4 (0.31) | 13.5 (0.31) | 1.90 [1.79, 2.01] | 12.4% | **-** | **-** |
|  | Oxford | Wegener et al. [23] | 20 | 13.1 (25.0) | 10.6 (28.57) | 2.49 [-14.15, 19.13] | 0.0% | **-** | **-** |
|  | Combined | Pooled effect | 918 | - | - | 1.54 [1.27, 1.80] | 100.0% | 11.40 (P < 0.00001) | 99% |
| Single support (%) | Sports | Lythgo et al. [7] 5 yrs | 69 | 42.7 (0.15) | 43.9 (0.10) | -1.20 [-1.24, -1.16] | 12.5% | **-** | **-** |
|  | Sports | Lythgo et al. [7] 6 yrs | 140 | 43.2 (0.15) | 44.0 (0.10) | -0.80 [-0.83, -0.77] | 12.6% | **-** | **-** |
|  | Sports | Lythgo et al. [7] 7 yrs | 152 | 43.2 (0.20) | 43.8 (0.20) | -0.60 [-0.65, -0.55] | 12.5% | **-** | **-** |
|  | Sports | Lythgo et al. [7] 8 yrs | 145 | 43.1 (0.20) | 43.9 (0.15) | -0.80 [-0.84, -0.76] | 12.5% | **-** | **-** |
|  | Sports | Lythgo et al. [7] 9 yrs | 117 | 42.9 (0.15) | 43.5 (0.15) | -0.60 [-0.64, -0.56] | 12.5% | **-** | **-** |
|  | Sports | Lythgo et al. [7] 10 yrs | 108 | 43.0 (0.15) | 43.6 (0.15) | -0.60 [-0.64, -0.56] | 12.5% | **-** | **-** |
|  | Sports | Lythgo et al. [7] 11 yrs | 105 | 42.3 (0.15) | 43.0 (0.10) | -0.70 [-0.74, -0.66] | 12.6% | **-** | **-** |
|  | Sports | Lythgo et al. [7] 12 yrs | 62 | 42.2 (0.20) | 43.2 (0.15) | -1.00 [-1.06, -0.94] | 12.3% | **-** | **-** |
|  | Sports | Pooled effect | 898 | - | - | -0.79 [-0.92, -0.65] | 100.0% | 11.26 (P < 0.00001) | 99% |
| Stance time (%) | Sports | Lythgo et al. [7] 5 yrs | 69 | 57.3 (0.26) | 56.1 (0.20) | 1.20 [1.12, 1.28] | 11.9% | **-** | **-** |
|  | Sports | Lythgo et al. [7] 6 yrs | 140 | 56.7 (0.15) | 55.8 (0.10) | 0.90 [0.87, 0.93] | 12.5% | **-** | **-** |
|  | Sports | Lythgo et al. [7] 7 yrs | 152 | 56.7 (0.15) | 56.1 (0.10) | 0.60 [0.57, 0.63] | 12.5% | **-** | **-** |
|  | Sports | Lythgo et al. [7] 8 yrs | 145 | 56.9 (0.15) | 56.2 (0.10) | 0.70 [0.67, 0.73] | 12.5% | **-** | **-** |
|  | Sports | Lythgo et al. [7] 9 yrs | 117 | 57.2 (0.15) | 56.5 (0.15) | 0.70 [0.66, 0.74] | 12.4% | **-** | **-** |
|  | Sports | Lythgo et al. [7] 10 yrs | 108 | 57.0 (0.15) | 56.4 (0.15) | 0.60 [0.56, 0.64] | 12.4% | **-** | **-** |
|  | Sports | Lythgo et al. [7] 11 yrs | 105 | 57.7 (0.20) | 56.9 (0.15) | 0.80 [0.75, 0.85] | 12.3% | **-** | **-** |
|  | Sports | Lythgo et al. [7] 12 yrs | 62 | 57.8 (0.20) | 56.8 (0.15) | 1.00 [0.94, 1.06] | 12.1% | **-** | **-** |
|  | Unknown | Moreno-Hernandez et al.[10] | 61 | 57.04 (3.03) | 56.3 (1.62) | 0.74 [-0.12, 1.60] | 1.5% | **-** | **-** |
|  | Combined | Pooled effect | 959 | - | - | 0.81 [0.70, 0.92] | 100.0% | 14.24 (P < 0.00001) | 98% |
| Swing time (%) | Unknown | Moreno-Hernandez et al.[10] | 61 | 42.97 (3.04) | 43.71 (1.62) | -0.74 [-1.60, 0.12] | 100.0% | 1.68 (P = 0.09) | N/A |
| Contact time (ms) | Walking | Kristen et al. [15] | 30 | 640.0 (125.0) | 591.0 (107.0) | 49.00 [-9.88, 107.88] | 100% | 1.63 (P = 0.10) | N/A |
| Angle of gait (°) | Sports | Lythgo et al. [7] 5 yrs | 69 | 1.0 (0.51) | 0.1 ( 0.56) | 0.90 [0.72, 1.08] | 12.3% | **-** | **-** |
|  | Sports | Lythgo et al. [7] 6 yrs | 140 | 1.4 (0.41) | 1.4 (0.46) | 0.00 [-0.10, 0.10] | 12.6% | **-** | **-** |
|  | Sports | Lythgo et al. [7] 7 yrs | 152 | 1.9 (0.41) | 2.5 (0.46) | -0.60 [-0.70, -0.50] | 12.7% | **-** | **-** |
|  | Sports | Lythgo et al. [7] 8 yrs | 145 | 1.6 (0.36) | 1.9 (0.36) | -0.30 [-0.38, -0.22] | 12.7% | **-** | **-** |
|  | Sports | Lythgo et al. [7] 9 yrs | 117 | 2.3 (0.46) | 3.0 (0.51) | -0.70 [-0.82, -0.58] | 12.6% | **-** | **-** |
|  | Sports | Lythgo et al. [7] 10 yrs | 108 | 2.4 (0.51) | 2.5 (0.71) | -0.10 [-0.27, 0.07] | 12.4% | **-** | **-** |
|  | Sports | Lythgo et al. [7] 11 yrs | 105 | 2.9 (0.51) | 2.5 (0.56) | 0.40 [0.25, 0.55] | 12.5% | **-** | **-** |
|  | Sports | Lythgo et al. [7] 12 yrs | 62 | 2.5 (0.56) | 2.3 (0.61) | 0.20 [-0.01, 0.41] | 12.2% | **-** | **-** |
|  | Walking | Wolf et al. [8] | 18 | 11.5 (21.21) | 14.6 (18.24) | -3.10 [-16.02, 9.82] | 0.1% | **-** | **-** |
|  | Combined | Pooled effect | 916 | - | - | -0.03 [-0.35, 0.29] | 100.0% | 0.19 (P = 0.85) | 98% |
|  | Walking (greater flexibility) | Wolf et al. [8] | 18 | 12.1 (5.1) | 14.6 (4.3) | -2.50 [-5.58, 0.58] | 100.0% | 1.59 (P = 0.11) | N/A |
| Progression angle (°) | Oxford | Wilkinson et al. [20] | 31 | 4.5 (8.8) | 7.0 (10.5) | -2.50 [-7.32, 2.32] | 100.0% | 1.02 (P=0.31) | N/A |
|  | Athletic | Wilkinson et al. [20] | 30 | 6.6 (8.5) | 7.0 (10.5) | -0.40 [-5.19, 4.39] | 100.0% | 0.16 (P=0.87) | N/A |

A negative mean difference value indicates a decrease during shod walking compared to barefoot walking. N/A indicates not applicable.
