# Supplementary material for: Effect of children's shoes on gait: a systematic review and meta-analysis
Source: J Foot Ankle Res. 2011 Jan 18;4:3. doi: 10.1186/1757-1146-4-3 (PMC3031211; doi:10.1186/1757-1146-4-3)
Supplement: Additional file 2 — Spatio-temporal variables for barefoot and shod running. [file 1757-1146-4-3-S2.DOC]

**Additional File 2:** Mean differences and statistical significance for spatio-temporal variables for shod and barefoot running.

| **Variable** | **Shoe Condition** | **Authors** | **n** | **Shod: mean(SD)** | **Barefoot: mean(SD)** | **Mean difference [95%CI]** | **Weighting** | **Statistical significance: z Score (P)** | **Heterogeneity: *I*^2^%** |
| --- | --- | --- | --- | --- | --- | --- | --- | --- | --- |
| Running velocity (m/s) | Unknown | Lieberman et al. [25] | 17 | 4.9 (0.5) | 5.1(0.5) | -0.20 [-0.54, 0.14] | 100.0% | 1.17 (P = 0.24) | N/A |
| Sprinting velocity (m/s) | Unknown | Tazuke [26] | 4 | 4.98 (0.46) | 5.14 (0.42) | -0.16 [-0.77, 0.45] | 100.0% | 0.52 (P = 0.60) | N/A |

A negative mean difference value indicates a decrease during shod running compared to barefoot running. N/A indicates not applicable
