# Supplementary material for: Effect of children's shoes on gait: a systematic review and meta-analysis
Source: J Foot Ankle Res. 2011 Jan 18;4:3. doi: 10.1186/1757-1146-4-3 (PMC3031211; doi:10.1186/1757-1146-4-3)
Supplement: Additional file 3 — Kinematic variables for barefoot and shod walking. [file 1757-1146-4-3-S3.DOC]

**Additional File 3:** Mean differences and statistical significance for kinematic variables for shod and barefoot walking.

| **Variable** | **Shoe Condition** | **Authors** | **n** | **Shod: mean(SD)** | **Barefoot: mean(SD)** | **Mean difference: [95%CI]** | **Weighting** | **Statistical significance:**  **z Score (P)** | **Heterogeneity: *I*^2^%** |
| --- | --- | --- | --- | --- | --- | --- | --- | --- | --- |
| Sagittal tibia-foot ROM (º) | Oxford | Wilkinson et al.[20] | 27 | 31.7 (5.4) | 25.3 (6.6) | 6.40 [3.40, 9.40] | 49.3% | - | - |
|  | Walking | Wolf et al. [8] | 18 | 29.2 (3.7) | 30.0 (4.6) | -0.80 [-3.53, 1.93] | 50.4% | - | - |
|  | Combined | Pooled effect | 45 | - | - | 2.75 [-4.31, 9.80] | 100.0% | 0.76 (P=0.45) | 91% |
|  | Athletic | Wilkinson et al.[20] | 26 | 32.9 (6.3) | 25.3 (6.6) | 7.60 [4.13, 11.07] | 100.0% | 4.29 (P < 0.0001) | N/A |
|  | Walking (increased flexibility) | Wolf et al. [8] | 18 | 29.0 (4.0) | 30.0 (4.6) | -1.00 [-3.82, 1.82] | 100.0% | 0.70 (P = 0.49) | N/A |
| Sagittal tibia-rearfoot ROM (º) | Oxford | Wegener et al. [23] | 20 | 24.51 (4.98) | 23.27 (4.82) | 1.24 [-1.80, 4.28] | 43.5% | - | - |
|  | Walking | Wolf et al. [8] | 18 | 26.6 (3.2) | 22.5 (3.7) | 4.10 [1.84, 6.36] | 56.5% | - | - |
|  | Combined | Pooled effect | 38 | - | - | 2.86 [0.08, 5.64] | 100.0% | 2.01 (P = 0.04) | 54% |
|  | Walking (increased flexibility) | Wolf et al. [8] | 18 | 25.7(3.3) | 22.5(3.7) | 3.20 [0.91, 5.49] | 100.0% | 2.74 (P = 0.006) | N/A |
| Medial arch length ROM (º) | Walking | Wolf et al. [8] | 18 | 5.9(1.5) | 9.9(2.5) | -4.00 [-5.35, -2.65] | 100.0% | 5.82 (P < 0.00001) | N/A |
|  | Walking (increased flexibility) | Wolf et al. [8] | 18 | 6.0(1.8) | 9.9(2.5) | -3.90 [-5.32, -2.48] | 100.0% | 5.37 (P < 0.00001) | N/A |
| Hallux flexion ROM (º) | Oxford | Wegener et al. [23] | 20 | 23.39(2.56) | 34.91(4.10) | -11.52 [-13.64, -9.40] | 64.5% | - | - |
|  | Walking | Wolf et al. [8] | 18 | 25.7(3.5) | 37.1(5.1) | -11.40 [-14.26, -8.54] | 35.5% | - | - |
|  | Combined | Pooled effect | 38 | - | - | -11.48 [-13.18, -9.78] | 100.0% | 13.22 (P < 0.00001) | 0% |
|  | Walking (increased flexibility) | Wolf et al. [8] | 18 | 27.8(4.0) | 37.1(5.1) | -9.30 [-12.29, -6.31] | 100.0% | 6.09 (P < 0.00001) | N/A |
| ‘Subtalar’ rotation ROM (º) | Walking | Wolf et al. [8] | 18 | 7.4(1.7) | 6.5(1.3) | 0.90 [-0.09, 1.89] | 100.0% | 1.78 (P = 0.07) | N/A |
|  | Walking (increased flexibility) | Wolf et al. [8] | 18 | 7.6(1.7) | 6.5(1.3) | 1.10 [0.11, 2.09] | 100.0% | 2.18 (P = 0.03) | N/A |
| Foot torsion ROM (º) | Walking | Wolf et al. [8] | 18 | 4.7(1.6) | 9.8(3.0) | -5.10 [-6.67, -3.53] | 100.0% | 6.36 (P < 0.00001) | N/A |
|  | Walking (increased flexibility) | Wolf et al. [8] | 18 | 5.2(2.0) | 9.8(3.0) | -4.60 [-6.27, -2.93] | 100.0% | 5.41 (P < 0.00001) | N/A |
| Forefoot supination ROM (º) | Walking | Wolf et al. [8] | 18 | 6.5(2.1) | 8.4(2.7) | -1.90 [-3.48, -0.32] | 100.0% | 2.36 (P = 0.02) | N/A |
|  | Walking (increased flexibility) | Wolf et al. [8] | 18 | 6.5(1.8) | 8.4(2.7) | -1.90 [-3.40, -0.40] | 100.0% | 2.48 (P = 0.01) | N/A |
| Foot rotation ROM (º) | Walking | Wolf et al. [8] | 18 | 18.7 (4.3) | 20.9(3.9) | -2.20 [-4.88, 0.48] | 100.0% | 1.61 (P = 0.11) | N/A |
|  | Walking (increased flexibility) | Wolf et al. [8] | 18 | 19.4 (4.7) | 20.9(3.9) | -1.50 [-4.32, 1.32] | 100.0% | 1.04 (P = 0.30) | N/A |
| Forefoot width ROM (%) | Walking | Wolf et al. [8] | 18 | 4.3 (1.4) | 9.7(3.1) | -5.40 [-6.97, -3.83] | 100.0% | 6.74 (P < 0.00001) | N/A |
|  | Walking (increased flexibility) | Wolf et al. [8] | 18 | 5.9 (1.4) | 9.7(3.1) | -3.80 [-5.37, -2.23] | 100.0% | 4.74 (P < 0.00001) | N/A |
| Midfoot sagittal plane ROM (º) | Oxford | Wegener et al.[23] | 20 | 14.37 (7.20) | 21.81(4.47) | -7.44 [-11.15, -3.73] | 100.0% | 3.93 (P < 0.0001) | N/A |
| Midfoot frontal plane ROM (º) | Oxford | Wegener et al. [23] | 20 | 5.90 (3.45) | 8.97(2.88) | -3.07 [-5.04, -1.10] | 100.0% | 3.06 (P = 0.002) | N/A |
| Midfoot transverse plane ROM (º) | Oxford | Wegener et al. [23] | 20 | 5.82 (2.15) | 10.84(2.77) | -5.01 [-6.55, -3.48] | 100.0% | 6.39 (P < 0.00001) | N/A |
| Ankle frontal plane ROM (º) | Oxford | Wegener et al. [23] | 20 | 10.63 (4.62) | 12.32(3.66) | -1.68 [-4.27, 0.90] | 100.0% | 1.28 (P = 0.20) | N/A |
| Ankle transverse plane ROM (º) | Oxford | Wegener et al. [23] | 20 | 13.20 (4.77) | 12.81(4.60) | 0.39 [-2.52, 3.29] | 100.0% | 0.26 (P = 0.79) | N/A |
| Knee sagittal plane ROM (º) | Oxford | Wegener et al. [23] | 20 | 44.73 (10.36) | 35.52(8.93) | 9.21 [3.22, 15.21] | 100.0% | 3.01 (P = 0.003) | N/A |
| Knee frontal plane ROM (º) | Oxford | Wegener et al. [23] | 20 | 6.28 (2.34) | 6.26(2.50) | 0.02 [-1.48, 1.52] | 100.0% | 0.02 (P = 0.98) | N/A |
| Knee transverse plane ROM (º) | Oxford | Wegener et al. [23] | 20 | 20.78 (7.79) | 20.91(7.28) | -0.13 [-4.80, 4.55] | 100.0% | 0.05 (P = 0.96) | N/A |
| Hip sagittal plane ROM (º) | Oxford | Wegener et al. [23] | 20 | 45.19 (4.67) | 43.15(5.76) | 2.04 [-1.21, 5.29] | 100.0% | 1.23 (P = 0.22) | N/A |
| Hip frontal plane ROM (º) | Oxford | Wegener et al. [23] | 20 | 12.53 (3.22) | 12.93(3.18) | -0.40 [-2.39, 1.58] | 100.0% | 0.40 (P = 0.69) | N/A |
| Hip transverse plane ROM (º) | Oxford | Wegener et al. [23] | 20 | 11.99 (3.39) | 10.89(3.56) | 1.10 [-1.05, 3.25] | 100.0% | 1.00 (P = 0.32) | N/A |
| Ankle max dorsiflexion (º) | Oxford | Wilkinson et al.[20] | 27 | 102.7 (7.8) | 109.9 (8.2) | -7.20 [-11.18, -3.22] | 100.0% | 3.54 (P=0.0004) | N/A |
|  | Athletic | Wilkinson et al.[20] | 26 | 108.2 (6.7) | 109.9 (8.2) | -1.70 [-5.45, 2.05] | 100.0% | 0.89 (P=0.37) | N/A |
| Ankle angle at foot lift (º) | Oxford | Wilkinson et al.[20] | 27 | 105.3 (9.2) | 111.0 (8.6) | -5.70 [-10.45, -0.95] | 100.0% | 2.35 (P=0.02) | N/A |
|  | Athletic | Wilkinson et al.[20] | 26 | 109.5 (7.8) | 111.0 (8.6) | -1.50 [-5.92, 2.92] | 100.0% | 0.67 (P=0.51) | N/A |
| Ankle max plantarflexion (º) | Oxford | Wilkinson et al.[20] | 27 | 134.5 (10.5) | 135.2 (9.1) | -0.70 [-5.94, 4.54] | 100.0% | 0.26 (P=0.79) | N/A |
|  | Athletic | Wilkinson et al.[20] | 26 | 141.0 (6.4) | 135.2 (9.1) | 5.80 [1.58, 10.02] | 100.0% | 2.69 (P=0.007) | N/A |
| Ankle ROM, foot lift to max plantarflexion (º) | Oxford | Wilkinson et al.[20] | 27 | 29.2 (5.0) | 24.2 (6.9) | 5.00 [1.79, 8.21] | 100.0% | 3.05 (P=0.002) | N/A |
|  | Athletic | Wilkinson et al.[20] | 26 | 31.5 (7.0) | 24.2 (6.9) | 7.30 [3.56, 11.04] | 100.0% | 3.82 (P=0.0001) | N/A |

A negative mean difference value indicates a decrease during shod walking compared to barefoot walking. N/A indicates not applicable.
