# Supplementary material for: Effect of children's shoes on gait: a systematic review and meta-analysis
Source: J Foot Ankle Res. 2011 Jan 18;4:3. doi: 10.1186/1757-1146-4-3 (PMC3031211; doi:10.1186/1757-1146-4-3)
Supplement: Additional file 4 — Kinematic variables for barefoot and shod running. [file 1757-1146-4-3-S4.DOC]

**Additional File 4:** Mean differences and statistical significance for kinematic variables for shod and barefoot running.

| **Variable** | **Shoe Condition** | **Authors** | **n** | **Shod:**  **mean(SD)** | **Barefoot: mean(SD)** | **Mean difference: [95%CI]** | **Weighting** | **Statistical significance:**  **z Score (P)** | **Heterogeneity: *I*^2^%** |
| --- | --- | --- | --- | --- | --- | --- | --- | --- | --- |
| Ankle angle at foot strike (º) | Unknown | Lieberman et al. [25] | 17 | -2.7(9.0) | 4.1(10.9) | -6.80 [-13.52, -0.08] | 100.0% | 1.98 (P = 0.049) | N/A |
| Plantar foot angle at foot strike (º) | Unknown | Lieberman et al. [25] | 17 | -19.8(10.3) | -10.1(9.7) | -9.70 [-16.43, -2.97] | 100.0% | 2.83 (P = 0.005) | N/A |
| Knee angle at foot strike (º) | Unknown | Lieberman et al. [25] | 17 | 18.4(6.6) | 18.9(6.5) | -0.50 [-4.90, 3.90] | 100.0% | 0.22 (P = 0.82) | N/A |
| Knee lift angle (º) | Unknown | Tazuke [26] | 4 | 54.76(11.13) | 55.97(10.58) | -1.20 [-16.25, 13.84] | 100.0% | 0.16 (P = 0.88) | N/A |
| Knee angular velocity (º/s) | Unknown | Tazuke [26] | 4 | 1164.91(138.16) | 1325.49(49.30) | -160.59 [-304.34, -16.83] | 100.0% | 2.19 (P = 0.03) | N/A |
| Swing-back velocity (º/s) | Unknown | Tazuke [26] | 4 | 275.58(51.33) | 359.82(55.94) | -84.24 [-158.64, -9.84] | 100.0% | 2.22 (P = 0.03) | N/A |

A negative mean difference value indicates a decrease during shod running compared to barefoot running. N/A indicates not applicable
