# Supplementary material for: Effect of children's shoes on gait: a systematic review and meta-analysis
Source: J Foot Ankle Res. 2011 Jan 18;4:3. doi: 10.1186/1757-1146-4-3 (PMC3031211; doi:10.1186/1757-1146-4-3)
Supplement: Additional file 5 — Kinetic variables for barefoot and shod walking. [file 1757-1146-4-3-S5.DOC]

**Additional File 5:** Mean differences and statistical significance for kinetic variables for shod and barefoot walking.

| **Variable** | **Shoe Condition** | **Authors** | **n** | **Shod: mean(SD)** | **Barefoot: mean(SD)** | **Mean difference: [95%CI]** | **Weighting** | **Statistical significance: z Score(P)** | **Heterogeneity: *I*^2^%** |
| --- | --- | --- | --- | --- | --- | --- | --- | --- | --- |
| Vertical ground reaction force (%BW) | Walking | Kristen et al. [15] | 30 | 126.0 (19.5) | 119.7 (16.4) | 6.30 [-2.82, 15.42] | 100.0% | 1.35 (P = 0.18) | N/A |
| Anterior Posterior Max GRF (%BW) | Walking | Kristen et al. [15] | 30 | 19.8 (5.3) | 20.7 (5.6) | -0.90 [-3.66, 1.86] | 100.0% | 0.64 (P = 0.52) | N/A |
| Anterior Posterior Min GRF (%BW) | Walking | Kristen et al. [15] | 30 | 21.6 (10.3) | 22.6 (9.4) | -1.00 [-5.99, 3.99] | 100.0% | 0.39 (P = 0.69) | N/A |

A negative mean difference value indicates a decrease during shod walking compared to barefoot walking. N/A indicates not applicable
