# Supplementary material for: Effect of children's shoes on gait: a systematic review and meta-analysis
Source: J Foot Ankle Res. 2011 Jan 18;4:3. doi: 10.1186/1757-1146-4-3 (PMC3031211; doi:10.1186/1757-1146-4-3)
Supplement: Additional file 6 — Kinetic variables for barefoot and shod running. [file 1757-1146-4-3-S6.DOC]

**Additional File 6:** Mean differences and statistical significance for kinetic variables for shod and barefoot running.

| **Variable** | **Shoe Condition** | **Authors** | **n** | **Shod: mean(SD)** | **Barefoot: mean(SD)** | **Mean difference [95%CI]** | **Weighting** | **Statistical significance: z Score (P)** | **Heterogeneity: *I*^2^%** |
| --- | --- | --- | --- | --- | --- | --- | --- | --- | --- |
| Max vertical impact force (BW) | Sports | Alcantara et al. [21] (girls) | 4 | 1.59 (0.1) | 1.91 (0.01) | -0.32 [-0.42, -0.22] | 49.4% | - | - |
|  | Sports | Alcantara et al. [21] (boys) | 4 | 1.49 (0.06) | 1.44 (0.01) | 0.05 [-0.01, 0.11] | 50.6% | - | - |
|  | Sports | Pooled effect | 8 | - | - | -0.13 [-0.50, 0.23] | 100.0% | 0.72 (P = 0.47) | 97% |
|  | Walking | Alcantara et al. [21] (girls) | 4 | 1.28 (0.06) | 1.44 (0.01) | -0.16 [-0.22, -0.10] | 49.9% | - | - |
|  | Walking | Alcantara et al. [21] (boys) | 4 | 1.23 (0.05) | 1.91 (0.01) | -0.68 [-0.73, -0.63] | 50.1% | - | - |
|  | Walking | Pooled effect | 8 | - | - | -0.42 [-0.93, 0.09] | 100.0% | 1.62 (P = 0.11) | 99% |
| Rate of load at impact (BW/s) | Sports | Alcantara et al. [21] (girls) | 4 | 56.91 (3.46) | 196.62 (22.07) | -139.71 [-161.60, -117.82] | 49.5% | - | - |
|  | Sports | Alcantara et al. [21] (boys) | 4 | 41.69 (2.01) | 85.33 (12.62) | -43.64 [-56.16, -31.12] | 50.5% | - | - |
|  | Sports | Pooled effect | 8 | - | - | -91.24 [-185.38, 2.90] | 100.0% | 1.90 (P = 0.06) | 98% |
|  | Walking | Alcantara et al. [21] (girls) | 4 | 49.99 (4.33) | 196.62 (22.07) | -146.63 [-168.67, -124.59] | 49.6% | - | - |
|  | Walking | Alcantara et al. [21] (boys) | 4 | 43.45 (2.41) | 85.33 (12.62) | -41.88 [-54.47, -29.29] | 50.4% | - | - |
|  | Walking | Pooled effect | 8 | - | - | -93.85 [-196.50, 8.80] | 100.0% | 1.79 (P = 0.07) | 98% |
| Long axis max tibial acceleration (g) | Sports | Alcantara et al. [21] (girls) | 4 | 4.36 (0.27) | 6.52 (0.37) | -2.16 [-2.61, -1.71] | 49.9% | - | - |
|  | Sports | Alcantara et al. [21] (boys) | 4 | 3.38 (0.14) | 4.32 (0.42) | -0.94 [-1.37, -0.51] | 50.1% | - | - |
|  | Sports | Pooled effect | 8 | - | - | -1.55 [-2.74, -0.35] | 100.0% | 2.54 (P = 0.01) | 93% |
|  | Walking | Alcantara et al. [21] (girls) | 4 | 3.87 (0.3) | 6.52 (0.37) | -2.65 [-3.12, -2.18] | 49.7% | - | - |
|  | Walking | Alcantara et al. [21] (boys) | 4 | 2.65 (0.16) | 4.32 (0.42) | -1.67 [-2.11, -1.23] | 50.3% | - | - |
|  | Walking | Pooled effect | 8 | - | - | -2.16 [-3.12, -1.20] | 100.0% | 4.40 (P <0.0001) | 89% |
| Rate of tibia acceleration (g/s) | Sports | Alcantara et al. [21] (girls) | 4 | 172.29 (14.54) | 424.88 (37.72) | -252.59 [-292.21, -212.97] | 50.6% | - | - |
|  | Sports | Alcantara et al. [21] (boys) | 4 | 121.39 (7.77) | 256.56 (46.99) | -135.17 [-181.84, -88.50] | 49.4% | - | - |
|  | Sports | Pooled effect | 8 | - | - | -194.56 [-309.62, -79.49] | 100.0% | 3.31 (P =0.0009) | 93% |
|  | Walking | Alcantara et al. [21] (girls) | 4 | 163.25 (18.67) | 424.88 (37.72) | -261.63 [-302.88, -220.38] | 56.4% | - | - |
|  | Walking | Alcantara et al. [21] (boys) | 4 | 110.73 (9.09) | 256.56 (46.99) | -145.83 [-192.73, -98.93] | 43.6% | - | - |
|  | Walking | Pooled effect | 8 | - | - | -211.13 [-242.11, -180.16] | 100.0% | 13.36 (P < 0.00001) | 92% |
| Shock wave transmission as a ratio of maximum acceleration (g/BW) | Sports | Alcantara et al. [21] (girls) | 4 | 3.06 (0.2) | 3.41 (0.11) | -0.35 [-0.57, -0.13] | 54.8% | - | - |
|  | Sports | Alcantara et al. [21] (boys) | 4 | 2.45(0.16) | 3.04 (0.22) | -0.59 [-0.86, -0.32] | 45.2% | - | - |
|  | Sports | Pooled effect | 8 | - | - | -0.46 [-0.69, -0.22] | 100.0% | 3.84 (P = 0.0001) | 45% |
|  | Walking | Alcantara et al. [21] (girls) | 4 | 3.27 (0.24) | 3.41 (0.11) | -0.14 [-0.40, 0.12] | 50.1% | - | - |
|  | Walking | Alcantara et al. [21] (boys) | 4 | 2.26 (0.16) | 3.04 (0.22) | -0.78 [-1.05, -0.51] | 49.9% | - | - |
|  | Walking | Pooled effect | 8 | - | - | -0.46 [-1.09, 0.17] | 100.0% | 1.43 (P = 0.15) | 91% |

A negative mean difference value indicates a decrease during shod running compared to barefoot running.
